# Associations between a Leader's Work Passion and an Employee's Work Passion: A Moderated Mediation Model

**DOI:** 10.3389/fpsyg.2017.01447

**Published:** 2017-08-28

**Authors:** Jingjing Li, Jian Zhang, Zhiguo Yang

**Affiliations:** ^1^Donlinks School of Economics and Management, University of Science and Technology Beijing Beijing, China; ^2^Laboratory of Talent Evaluation of Land and Resources Beijing, China; ^3^Alumni Association and Foundation Office, University of Science and Technology Beijing Beijing, China

**Keywords:** work passion, moderated mediation model, emotional contagion, goal content, Johnson-Neyman

## Abstract

Based on the theory of emotional contagion and goal content, this study explored the positive associations between a leader's work passion and employees' work passion. This study investigated 364 employees and their immediate leaders from China, constructed a moderated mediation model, and used SPSS-PROCESS in conjunction with the Johnson-Neyman technique to analyze the data. The results showed that a leader's work passion was transferred to employees via emotional contagion, and the contagion process was moderated by leader–employee goal content congruence. This study provides a potential way to stimulate employees' work passion from the perspective of leader–employee interactions. Moreover, the limitations of the study and potential topics for future research are discussed.

## Introduction

Work passion is a strong inclination toward work that people like, find important, and want to invest their time and energy in (Vallerand and Houlfort, 2003). Both organizational researchers and practitioners have noted the importance of work passion (Boyatzis et al., 2002; McDaniel et al., 2009). For example, work passion may enhance employees' positive affect experience (Forest et al., 2011; Donahue et al., 2012) and organizational citizenship behavior at work (Astakhova, 2015; Burke et al., 2015), and it may improve employees' work performance (Ho et al., 2011; Bélanger et al., 2013; Burke et al., 2015) as well as their well-being (Zigarmi et al., 2009; Birkeland, 2014), which is the target of organizational positive psychology research (Di Fabio and Palazzeschi, 2015; Di Fabio et al., 2016). However, employees generally lack work passion in reality. For example, a Chinese survey on work passion shows that only 2.5% of respondents consistently have work passion, and 47.2% do not have it (Chen, 2010). Fieldwork indicates that leaders typically have more work passion than their employees (Institute of Psychology Chinese Academy of Science, 2013). Therefore, it is important to the field of organizational psychology to consider whether a leader's work passion may be transmitted to employees. This is the first issue we aim to explore.

According to leadership research, there are trickle-down effects between a leader and employees (Aryee et al., 2007), which indicates that a leader's affect, trait, cognition, attitude, and behavior induce similar responses in followers or groups (Wang et al., 2015). Moreover, the trickle-down effect on affect occurs via emotional contagion (Bono and Ilies, 2006; Johnson, 2008). Therefore, a leader's work passion may be a source of employees' work passion and transferred to employees through emotional contagion. In addition, Cardon (2008) developed a conceptual model of entrepreneurial passion contagion, which claims that with emotional contagion process entrepreneurial passion is transferred to his or her employees. Since entrepreneurial passion is an entrepreneur's work passion in the entrepreneurial work context (Perttula and Cardon, 2011), we can speculate that a leader's work passion may also be transferred to employees through emotional contagion. This is the second issue we aim to verify.

In our field investigation, we have found that even if a group of employees follows the same passionate leader, some employees may be more stimulated and full of passion than other employees. Particularly in China, leaders are not good at displaying their passion. Thus, we have to explore moderating factors that promote the emotional contagion process. Previous studies mainly focus on the factors from either an emotional sender or receiver. However, the complete emotional contagion process relates to both individuals, and it is an interactive activity. Thus, we may obtain novel outcomes by considering sender and receiver factors, for example, the similarity between them (Hatfield et al., 2014), particularly the goal content congruence between them. The reason is that goal content is what individuals pursue (Deci and Ryan, 2000; Vansteenkiste et al., 2007), thus determining individuals' behavioral choices in the workplace (Janssen and Yperen, 2004). If the leader–employee goal content congruence is high, they will have more similarities, and the leader will be more willing to communicate with their employees; moreover, the employees will pay more attention to the leader. This leads to a higher degree of contagion (Stockert, 1994). Therefore, the third issue is to explore the moderating effect of leader–employee goal content congruence in the process of work passion contagion.

In summary, the main purpose of this research is to solve the problem of how to transfer a leader's work passion to employees as well as explain why only some employees have work passion. Its theoretical and practical contributions are mainly embodied in three aspects. First, this study confirms the trickle-down effect of work passion, which indicates that we have identified a new source of employees' work passion from their leader. Second, this study verifies the mediating effect of emotional contagion on the relationship between a leader's work passion and employee's work passion, which fills the research gap on the work passion transmission mechanism, and provides empirical support for the entrepreneurial passion contagion model. Third, an essential moderating factor, leader–employee goal content congruence, has been identified, which combines the fit theory and emotional contagion theory and provides a preventive intervention for transferring work passion.

## Theory and hypotheses

### Leader's work passion and employee's work passion

Employee work passion is a new concept in organizational psychology. Besides the definition provide by Vallerand et al. (2003), some researchers also give their definitions. For example, Zigarmi et al. (2009) suggest that employee work passion is an individual's persistent, emotionally positive, meaning-based, state of wellbeing stemming from reoccurring cognitive and affective appraisals of various job and organizational situations that result in consistent, constructive work intentions and behaviors. Perrewé et al. (2014) contend that work passion may be a higher order construct composed of other closely related constructs such as engagement, affect, desire, and thriving. In sum, most of the studies accept that work passion contains the affective and cognitive components, love to work and identification to work. Vallerand et al. have developed a robust measurement of work passion confirmed in many cultures (Marsh et al., 2013; Burke et al., 2015), and large achievements have obtained based on their work. So in this research we also use the definition of Vallerand et al. (2003). The majority of work passion research devotes significant attention to the issues of what and how individuals' work passion affects their own feelings and behaviors. In contrast, there is a lack of concern regarding the specific factors that promote an individual's work passion.

Research on the source of work passion is mainly composed of the following aspects: the internalization of the representation of work in an individual's identity (Vallerand et al., 2003); cognitive and affective evaluation of work and organization (Zigarmi et al., 2011); and engagement in special work activities (i.e., entrepreneurship) (Cardon et al., 2009). In summary, a leader's emotion, cognition, and behavior may represent organizational context factors that impact employees' work passion. For example, Zigarmi et al. have indicated that a leader's self-concern orientation results in employees' negative affect experience, which reduces employees' work passion; a leader's other-orientation exhibits a significant direct correlation with employees' positive job-specific affect, thus increasing employees' work passion (Zigarmi and Roberts, 2012). Furthermore, cognitive perception research suggests that leaders who act in an ethical manner (Permarupan et al., 2013), provide employees recognition (Permarupan et al., 2013), and maintain connectedness with their employees may promote an employee's work passion (Luo et al., 2014). As work passion is a concept with cognition, affect and motivation components (Perttula and Cardon, 2011), an exploration of the antecedent variables of employees' work passion from their leaders' work passion may provide more worthy outcomes.

In the field of entrepreneurial passion, a branch of passion research, it has been reported that entrepreneurial passion can enhance employees' work passion (Cardon, 2008). This study also notes that entrepreneurs are not always the transmitters of passion because they may have bounded emotionality or emotional suppression. Thus, it is necessary to identify the “emotional leader” of the organization (Cardon, 2008). Positive psychology highlights leaders' behavior as the main source for employees' positive emotional experience and psychological state (Dasborough and Ashkanasy, 2002; Bono and Ilies, 2006). Furthermore, there is a trickle-down effect between leaders and employees, which indicates that a leader's emotional state may elicit the same state of his/her employees. Combined with passion theory, a passionate leader prefers to display his or her passion positively and frequently and share his or her identification with work. Over time, employees begin to internalize this emotion, and they are also likely to experience work passion (Cardon, 2008).

H1: A leader's work passion positively relates to employee's work passion.

### The mediating role of emotional contagion

Emotional contagion is a process in which an individual or group influences another individual or group's affective state, behavior, and attitude through subconscious emotional mimicry or conscious social comparison (Barsade, 2002; Du et al., 2011). Many leadership studies indicate that leaders' positive affect relates to followers' positive affect at work via emotional contagion (Bono and Ilies, 2006; Johnson, 2008). This is called trickle-down effect, that a leader's affect and behavior can induce similar responses in followers or groups (Wang et al., 2015).

In addition, the other reason that this study selected emotional contagion as a mediating variable is the model of entrepreneurial passion contagion. This conceptual model suggests that entrepreneurial passion may be transferred to employees via emotional contagion. To be specific, a passionate entrepreneur tends to display his or her emotion more frequently and intensely. Individuals are born with the ability to mimic other individuals; thus, employees automatically and subconsciously mimic the entrepreneur's facial expressions as well as the entrepreneur's body language and movements. This mimicry process may evoke employees' positive emotion. In addition, work passion includes a cognitive element, namely, identification with work. A passionate entrepreneur conveys to employees the positive signal that he or she loves his or her work, the work is important, and the work has a good prospect. If a leader wants to impact employees' attitudes and behaviors and make them recognize the meaningfulness of their work, this process must occur through social comparison (Boyatzis et al., 2015). With social comparison, employees want to be a member of the elite and attempt to understand why the entrepreneur experiences work passion as well as determine whether they should share the same feelings of a deep identity connection with the work or organization. If an identity connection is present along with positive emotions, employees will also experience work passion (Cardon, 2008). Because entrepreneurial passion is passion in a particular work setting, the entrepreneurial context (Perttula and Cardon, 2011), work passion and entrepreneurial passion have the same components. Given that, we suggest the emotional contagion model may also be suitable for work passion transference process in general work settings. Furthermore, contact between emotional sender and receiver is the necessary condition of emotional contagion (Lars-Olov, 2006). And contact is more likely to occur between employees and their immediate leader than the entrepreneur. Therefore, we speculate that a leader's work passion may be transferred to employees via emotional contagion.

H2: Emotional contagion mediates the relationship between a leader's work passion and an employee's work passion.

### The moderating role of leader–employee goal content congruence

From a theoretical perspective, the idea that a leader's work passion may be transferred to employees through emotional contagion is possible. However, in our field investigation, we have found that despite following the same passionate leader, some employees are full of passion in contrast to other employees. This finding implies that some factors hinder emotional contagion. Thus, an exploration of the factors that result in differences in emotional contagion efficiency is the key to facilitating emotional contagion.

Emotional contagion is influenced by individual characteristics, situational factors and interpersonal relations. For example, emotional contagion occurs more easily for women than men and extroverts than introverts (Wang et al., 2010). Individuals in high-level power positions are more likely to share feelings with individuals in low-level positions. When the emotion sender and receiver have more similarities, they are more likely to mimic each other's behaviors, have closer relationships, and transmit emotions (Van Orden and Joiner, 2006). Individual characteristics and the leader-member relationship are relatively stable; thus, it is difficult to induce emotional contagion from these two aspects. Influenced by Confucianism, Chinese leaders are not good at displaying their passion. Interpersonal relations, referred to as Guanxi in China, are critical in Chinese organizations (Park and Luo, 2001; Chen and Peng, 2008); if the emotional sender and receiver have more similarities, they may have closer relations, and emotional contagion is easier to occur. Therefore, focusing on the similarity between emotional sender and receiver may obtain more practical and operational outcomes.

We have conducted a literature review from the person–supervisor fit perceptive. It includes the match between supervisors and subordinates (Van Vianen, 2000), leader–follower value congruence (Krishnan, 2002; Colbert, 2005), supervisor–subordinate personality similarity (Schaubroeck and Lam, 2002), and manager–employee goal congruence (Witt, 1998). Moreover, the results of a meta-analysis indicate that goals will result in effect sizes greater than personality-based measures and less than values-based measures (Kristof-brown et al., 2005). These studies substantially facilitate the identification of a more stable variable to explore the similarity between a leader and his or her employees. Moreover, in the workplace, goal content is the root variable that influences individuals' behaviors. It is referred to as work value orientation in workplace research (Vansteenkiste et al., 2007). Thus, it is a more stable variable in the workplace. Different goal content orientations lead to different behavioral choices (Janssen and Yperen, 2004). Kasser and Ryan (1996) distinguished intrinsic goals (self-acceptance, affiliation, community-feeling, and physical fitness) from extrinsic goals (financial success, social recognition, appealing appearance). Intrinsic goal pursuit engenders an inward orientation that is conducive to need satisfaction (needs for autonomy, competence, and relatedness) (Vansteenkiste et al., 2010), whereas extrinsic goal pursuit may engender an outward orientation that is focused on garnering self-worth through achievement and external validation, which thus detracts from basic need satisfaction (Vansteenkiste et al., 2010). According to the emotional contagion theory and fit theory, if the leader–employee goal content congruence is high, they will have more similarities, the leader will be more willing to communicate with employees, and employees will tend to mimic and accept the leader's contagion (Stockert, 1994). In contrast, if leader–employee goal content congruence is low, there will be more obvious divergence in choices and fewer close relationships; consequently, transmitting work passion via emotional contagion is difficult. Thus, the following hypothesis is proposed.

H3: Leader–employee goal congruence positively moderates the strength of the mediated relationship between a leader's work passion and employee's work passion via emotional contagion. Namely, the relationship is stronger when the leader–employee goal content congruence is high than when it is low.

**Figure 1:**
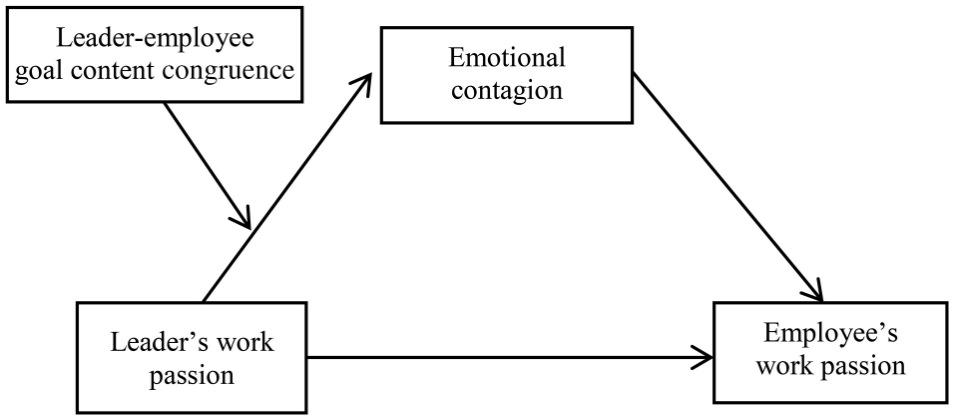
Conceptual model of the study.

We have developed a moderated mediation model by combining Hypotheses 1, 2, and 3, as shown in Figure 1. Specifically, the effect of a leader's work passion on an employee's work passion is mediated by emotional contagion, and the mediating effect is moderated by the leader–employee goal content congruence.

## Materials and methods

### Participants and procedures

The convenience sampling method was used to select 51 Chinese firms from Beijing, Hebei, Tianjin, and Shanxi Provinces to participate in our survey. We considered the diversity of the enterprise property, which mainly involved private enterprises, state-owned enterprises, and foreign-capital enterprises. The firms' human resource managers helped to collect the data from two sources: the employees and their immediate leaders. The employees filled out the questionnaires that measure their work passion, goal content, and emotional contagion. And the immediate leaders completed the questionnaires that measure leader's work passion and goal content. There were both printed and electronic versions of the questionnaires. The human resource managers distributed the printed questionnaires to the employees, who subsequently returned the completed questionnaires in envelopes. We sent the electronic questionnaires via e-mail to the participants, and they returned them after completion. We invited 423 participants, and 380 participants accepted our survey. We ultimately retained 364 paired samples after deleting the samples with incomplete information or non-direct relations between the leader and employees. The overall response rate was 86.05%. Among the leaders, 59.3% were male, 40.7% were female, and 90% were middle managers. Among the employees, 54.0% were male, and 46.0% were female. The mean age was 30.08 years (with a range from 20 to 59 years). The average tenure was 4.398 years (with a range from 1 to 25 years). A total of 44.2% of the employees had a bachelor's degree, and 15.9% had a master's degree or higher.

### Ethical statement

The study was reviewed and approved by the ethical committee of the University of Science and Technology Beijing. Written informed consent was obtained from all employees and their managers. All participants were informed of their right to withdraw from the survey at any time.

### Measures

#### Control variables

We controlled for demographic variables that have been found to be related to emotional contagion and work passion: gender, age, tenure, education, and job position.

#### Work passion

To assess leader and employee work passion, we used a 19-item scale developed by Vallerand and Houlfort (2003). The scale is appropriate for China (Burke et al., 2015). The passion scale is composed of two dimensions: harmonious passion (7 items, e.g., “my work is harmonious with other activities in my life”) and obsessive passion (7 items, e.g., “the urge is so strong, I can't help myself from doing my work”). It also contains five additional items as passion criteria to assess whether a participant has passion, such as “I invest much time doing the work.” Responses were recorded for all items on a 7-point agreement scale. The Cronbach's alpha for the total scale was 0.946. For the harmonious passion, obsessive passion, and passion criteria, the Cronbach's alpha coefficients were 0.871, 0.894, and 0.813, respectively, which indicates high reliability. In addition, a confirmatory factor analysis (CFA) was conducted, and the overall fit indexes of the scale were as follows: χ^2^*/df* = 2.412, *NFI* = 0.957, *RFI* = 0.933, *IFI* = 0.974, *TLI* = 0.960, *CFI* = 0.974, and *RMSEA* = 0.062. The CFA results show that the work passion scale has good construct validity.

#### Leader–employee goal content congruence

To assess the leader–employee goal content congruence, we used a 7-point, 35-item scale of the aspiration index developed by Kasser and Ryan (1996) and introduced it in the workplace. This scale has seven dimensions: financial success, social recognition, appealing appearance, self-acceptance, affiliation, community-feeling, and physical fitness (Kasser and Ryan, 1996). Financial success, appealing appearance and social recognition were classified as extrinsic goals (Kasser and Ryan, 1996), whereas self-acceptance, affiliation, community-feeling, and physical fitness were classified as intrinsic goals (Kasser and Ryan, 1996). Respondents were instructed to score all items according to their real feelings. We initially calculated the mean of the items as the measured value of the relevant goal content and subsequently calculated the Euclidean distance as the difference between a leader's goal content and employees' goal content as follows:

$$\begin{array}{l} \text{leader}-\text{employee goal content congruence}=-\text{d}\left(\text{LGC},\text{EGC}\right)\\ =-\text{sqrt}\left({\left(\text{LIGC}-\text{EIGC}\right)}^{2}+\left(\text{LEGC}-{\text{EEGC}}^{\wedge }2\right)\right) \end{array}$$

Note: LGC, leader goal content; EGC, employee goal content; LIGC, leader intrinsic goal content; EIGC, employee intrinsic goal content; LEGC, leader extrinsic goal content; EEGC, employee extrinsic goal content.

The Cronbach's alpha for the total scale was 0.957. For the intrinsic goal content and extrinsic goal content, the Cronbach's alpha coefficients were 0.928 and 0.922, respectively, which indicates high reliability. Furthermore, a CFA was conducted, and the overall fit indexes of the scale were as follows: χ^2^*/df* = 2.987, *NFI* = 0.989, *RFI* = 0.952, *IFI* = 0.992, *TLI* = 0.968, *CFI* = 0.992, and *RMSEA* = 0.074. The CFA results show that the goal content scale has good construct validity.

#### Emotional contagion

To assess emotional contagion, we based the measurement on the 5-point, 15-item scale of emotional contagion developed by Doherty (1997). We modified the measurement to make it suitable for the workplace. First, we omitted several items, such as “I sense my body responding when the one I love touches me” and “when I look into the eyes of the one I love, my mind is filled with thoughts of romance.” We subsequently put all items into the workplace. For example, the item “being with a happy person picks me up when I'm feeling down” was adjusted to “working together with a happy leader picks me up when I'm feeling down.” Finally, the respondents were instructed to score all items based on their real feelings toward their leader. A higher score was associated with more sufficient emotional contagion between the leader and employees. The Cronbach's alpha for the total scale was 0.929, which indicates high reliability. A CFA was conducted, and the overall fit indexes of the scale were as follows: χ^2^*/df* = 1.155, *NFI* = 0.910, *RFI* = 0.882, *IFI* = 0.987, *TLI* = 0.983, *CFI* = 0.987, and *RMSEA* = 0.040. The CFA results show that the emotional contagion scale has good construct validity.

### Data analysis

We initially used Amos 20.0 to examine the discriminant validity of the key variables in this research. The adequacy of the model was tested based on the χ^2^*/df, TLI, CFI, GFI, NFI, IFI*, and *RMSEA*. We subsequently presented the means, standard deviations, and correlations among the study variables. Following the two preliminary data analyses, we used SPSS-PROCSS to analyze the moderated mediation model. In addition, we used the Johnson-Neyman technique to determine in which regions of leader–employee goal content congruence the effect of leader's work passion on employees' work passion is significant and non-significant.

### Results

#### Discriminant validity of variables

A CFA of the research model was conducted through structural equation modeling using Amos 20.0. We compared a four-factor model (M0) with a three-factor model (M1), a two-factor model (M2), and a one-factor model (M4). In M0, we treated four variables (leader's work passion, employee's work passion, emotional contagion, and leader–employee goal content congruence) as four independent factors. In M1, we loaded the leader's work passion and employee's work passion items on one factor. In M2, we loaded employee's work passion, emotional contagion and leader–employee goal content congruence items on one factor. In M3, we loaded all variables on one factor. We tested the overall model fit via goodness-of-fit indexes, including χ^2^*/df, TLI, CFI, GFI, NFI, IFI*, and *RMSEA*. The results show that the four-factor model (M0) fits the data better than the other models (Table 1).

**Table 1:** Fit indices of models.

**Model**	**χ^2^**	**χ*^2^*/*df***	***TLI***	***CFI***	***GFI***	***NFI***	***IFI***	***RMSEA***
M0	45.992	1.5859	0.990	0.994	0.975	0.983	0.994	0.004
M1	1084.725	33.898	0.450	0.609	0.632	0.604	0.611	0.301
M2	810.833	23.848	0.618	0.712	0.731	0.704	0.713	0.251
M3	1597.067	45.630	0.254	0.42	0.531	0.417	0.422	0.351

*M0, LWP, EWP, EC, L-EGCC; M1, LWP+EWP, EC, L-EGCC; M2, LWP, EWP+EC+L-EGCC; M3, LWP+EWP+EC+L-EGCC*.

#### Descriptive statistics

The descriptive statistics and correlations among the variables are presented in Table 2. An inspection of the correlations reveals that leader's work passion is positive related to his or her gender (*r* = 0.146^**^, *p* < 0.01) and job position (*r* = 0.095^+^, *p* < 0.1), is negative related to his or her age (*r* = −0.105^*^, *p* < 0.05), and it is not significantly related to his or her tenure (*r* = −0.084, *p* > 0.05) and education (*r* = 0.088, *p* > 0.05). Employee's work passion is positive related to his or her gender (*r* = 0.170^**^, *p* < 0.01), education (*r* = 0.195^**^, *p* < 0.01), and job position (*r* = 0.152^**^, *p* < 0.01), is negative related to his or her age (*r* = −0.153^**^, *p* < 0.01) and tenure (*r* = −0.156^**^, *p* < 0.01). Emotional contagion has not presented significant relations with participants' demographic variables. Moreover, the results show that leader's work passion is positively related to employee's work passion (*r* = 0.152^**^, *p* < 0.01) and positively related to emotional contagion (*r* = 0.152^**^, *p* < 0.01). The results also indicate that emotional contagion is positively related to employee's work passion (*r* = 0.308^**^, *p* < 0.01). The correlations among leader–employee goal content congruence and the other three variables (leader's work passion, employee's work passion or emotional contagion) are not significant. Thus, this conceptual model and the collected data are suitable to conduct further data analysis.

**Table 2:** Means, standard deviations, and correlations.

**Variable**	***M***	***SD***	**1**	**2**	**3**	**4**	**5**	**6**	**7**	**8**	**9**	**10**	**11**	**12**	**13**	**14**
1. Gender (L)	0.593	0.492	1													
2. Age (L)	36.882	6.569	0.108^*^	1												
3. Tenure (L)	9.995	4.593	0.158^**^	0.905^**^	1											
4. Education (L)	2.220	0.494	−0.153^**^	−0.019	−0.109^*^	1										
5. Job position (L)	2.195	0.417	−0.029	0.611^**^	0.522^**^	−0.142^**^	1									
6. Gender (E)	0.540	0.499	0.160^**^	0.088	0.102	0.022	0.063	1								
7. Age (E)	30.077	5.628	0.047	0.235^**^	0.242^**^	−0.024	0.241^**^	0.071	1							
8. Tenure (E)	4.398	3.739	0.071	0.148^**^	0.120^*^	0.130^*^	0.030	−0.014	0.505^**^	1						
9. Education (E)	1.761	0.708	−0.066	0.002	0.077	−0.204^**^	0.205^**^	−0.071	−0.060	−0.273^**^	1					
10. Job position (E)	1.080	0.294	−0.023	0.077	0.068	−0.026	0.326^**^	0.026	0.052	0.029	0.160^*^	1				
11. LWP	5.061	1.110	0.146^**^	−0.105^*^	−0.084	0.088	0.095^+^	0.106^*^	−0.001	0.033	0.000	0.064	1			
12. EWP	4.578	1.129	0.004	0.042	0.079	0.020	0.191^**^	0.112^**^	−0.153^**^	−0.156^**^	0.170^**^	0.195^**^	0.152^**^	1		
13. EC	4.182	0.726	−0.079	0.001	−0.003	−0.002	0.089	−0.082	−0.016	−0.008	0.028	0.064	0.152^**^	0.308^**^	1	
14. L-EGCC	−1.561	1.137	−0.051	0.074	0.093	0.108^*^	0.015	−0.027	0.012	0.116^*^	0.094	0.084	0.034	0.099	−0.009	1

*LWP, leader's work passion; L-EGCC, Leader–employee goal content congruence; EC, emotional contagion; EWP, employee's work passion*.

** *p ≤ 0.01*,

* *p ≤ 0.05*,

+ *p ≤ 0.1*.

#### The mediating effect of emotional contagion between a leader's work passion and an employee's work passion

This study uses SPSS 21.0 to analyze the mediating role of emotional contagion between a leader's work passion and an employee's work passion.

Model 1 in Table 3 indicates that a leader's work passion had a significantly positive effect on an employee's work passion (β = 0.102^*^, *p* < 0.05), which thus supports H1. As shown in Model 2 and Model 3, the positive effect of a leader's work passion on an employee's work passion was not significant (β = 0.054, *p* > 0.05) after adding emotional contagion into the regression equation, and emotional contagion had a positive effect on employee's work passion (β = 0.442^***^, *p* < 0.001), which indicates that emotional contagion fully mediated the relationship between a leader's work passion and employee's work passion. Further analysis was conducted using SPSS-PROCESS to confirm the indirect effect of a leader's work passion on an employee's work passion via emotional contagion. The results show that with a formal two-tailed significance test, the indirect effect was significant (Sobel *z* = 2.472, *p* < *0.05*). Bootstrap estimation confirmed the Sobel test, with 5,000 bootstrap samples and a 95% confidence interval of 0.016–0.092 around the indirect effect not containing 0. Therefore, the indirect effect of a leader's work passion on an employee's work passion via emotional contagion was significant, and H1-2 was fully supported.

**Table 3:** Regression results for testing mediating role of emotional contagion.

**Variables and statistic**	**EC as dependent variable**	**EWP as dependent variable**	
	**Model 2**	**Model 1**	**Model 3**
Constant	3.658^***^	3.835^***^	2.218^**^
Gender (L)	−0.129	0.002	0.059
Age (L)	−0.003	−0.050^*^	−0.048^*^
Tenure (L)	0.003	0.067^*^	0.066^*^
Education (L)	−0.025	0.198	0.209^+^
Job position (L)	0.138	0.565^**^	0.504^**^
Gender (E)	−0.129^+^	0.223	0.280^*^
Age (E)	−0.004	−0.036^**^	−0.035^**^
Tenure (E)	0.001	−0.016	−0.016
Education (E)	−0.012	0.138	0.143^+^
Job position (E)	0.078	0.468^*^	0.433^*^
LWP	0.108^**^	0.102	0.054
EC			0.442^***^
*F*	1.632	5.714	8.651
*R*^2^	0.049	0.152	0.228

*LWP, Leader's work passion; L-EGCC, Leader–employee goal content congruence; EC, Emotional contagion; EWP, Employee's work passion*.

*** *p ≤ 0.001*,

** *p ≤ 0.01*,

* *p ≤ 0.05*,

+ *p ≤ 0.1*.

#### The moderating effect of leader–employee goal content congruence

Based on the moderated mediation model described by Preacher et al. (2007) and Hayes (2013), we used the sample mean and mean±1 *SD* to distinguish the different levels of leader–employee goal content congruence and conducted a bootstrapping test. We utilized a bootstrap sample of 5,000. Under the 95% confidence interval, the leader–employee goal content congruence moderated the mediation effect of emotional contagion on a leader's work passion and employee's work passion. The results are shown in Table 4.

**Table 4:** Regression results for testing moderated mediation of leader–employee goal content congruence.

**Variables and statistics**	**EC**	**Se**	***t***	***p***	**LLCI**	**ULCI**
Constant	4.110^***^	0.537	7.649	0.000	3.053	5.166
LWP	0.129^**^	0.039	3.323	0.001	0.053	0.206
L-EGCC	−0.022	0.044	−0.503	0.616	−0.108	0.064
LWP^*^L-EGCC	0.062^*^	0.027^*^	2.291	0.023	0.009	0.116
Gender (L)	−0.113	0.076	−1.482	0.140	−0.263	0.037
Age (L)	0.004	0.020	0.192	0.848	−0.036	0.044
Tenure (L)	−0.004	0.028	−0.133	0.894	−0.059	0.051
Education (L)	−0.034	0.094	−0.366	0.715	−0.219	0.151
Job position (L)	0.093	0.146	0.638	0.524	−0.193	0.379
Gender (E)	−0.139	0.080	−1.743	0.082	−0.023	0.017
Age (E)	−0.003	0.010	−0.310	0.757	−0.026	0.027
Tenure (E)	0.000	0.014	0.032	0.757	−0.023	0.017
Education (E)	−0.017	0.056	−0.302	0.763	−0.126	0.092
Job position (E)	0.092	0.144	0.638	0.524	−0.191	0.375
*R*^2^	0.063					
Δ*R*^2^	0.017					

*LWP, Leader's work passion; L-EGCC, Leader–employee goal content congruence*.

*** *p ≤ 0.001*,

** *p ≤ 0.01*,

* *p ≤ 0.05*,

*^+^p ≤ 0.1*.

More specifically, with low leader–employee goal content congruence, the results of the bootstrapping test showed that β = 0.026, *CI* (−0.016, 0.065), which contains 0, thus indicating the mediating role of emotional contagion is not significant. With high leader–employee goal content congruence, the results of the bootstrapping test showed that β = 0.088, *CI* (0.036, 0.162), which did not contain 0, thus indicating that the mediating role of emotional contagion is significant. With a mean level of leader–employee goal content congruence, the bootstrapping test results showed that β = 0.057, *CI* (0.022, 0.104), which did not contain 0, thus indicating that the mediating role of emotional contagion is significant. H3 is thus supported.

**Figure 2:**
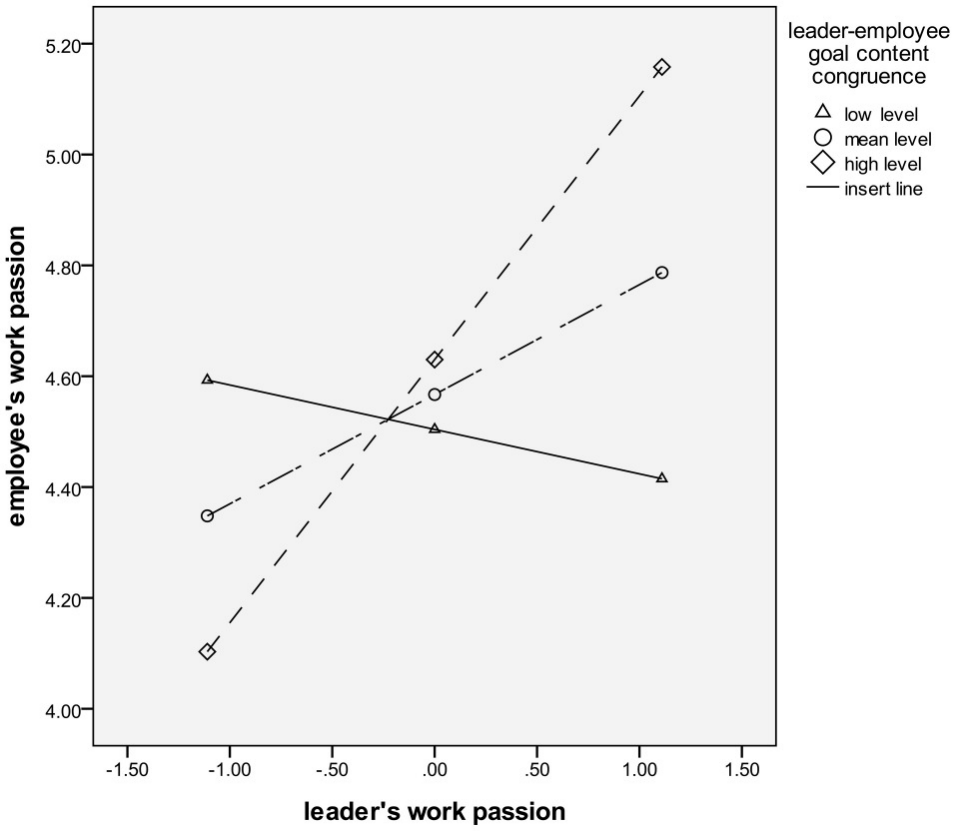
Moderation effect of leader–employee goal content congruence on the passion contagion process.

Figure 2 indicates that a leader's work passion has a negative effect on an employee's work passion when the leader–employee goal content congruence is at a low level (mean-1 *SD*). However, a leader's work passion has a significantly positive effect on an employee's work passion when the leader–employee goal content congruence is at a high level (mean+1 *SD*).

**Figure 3:**
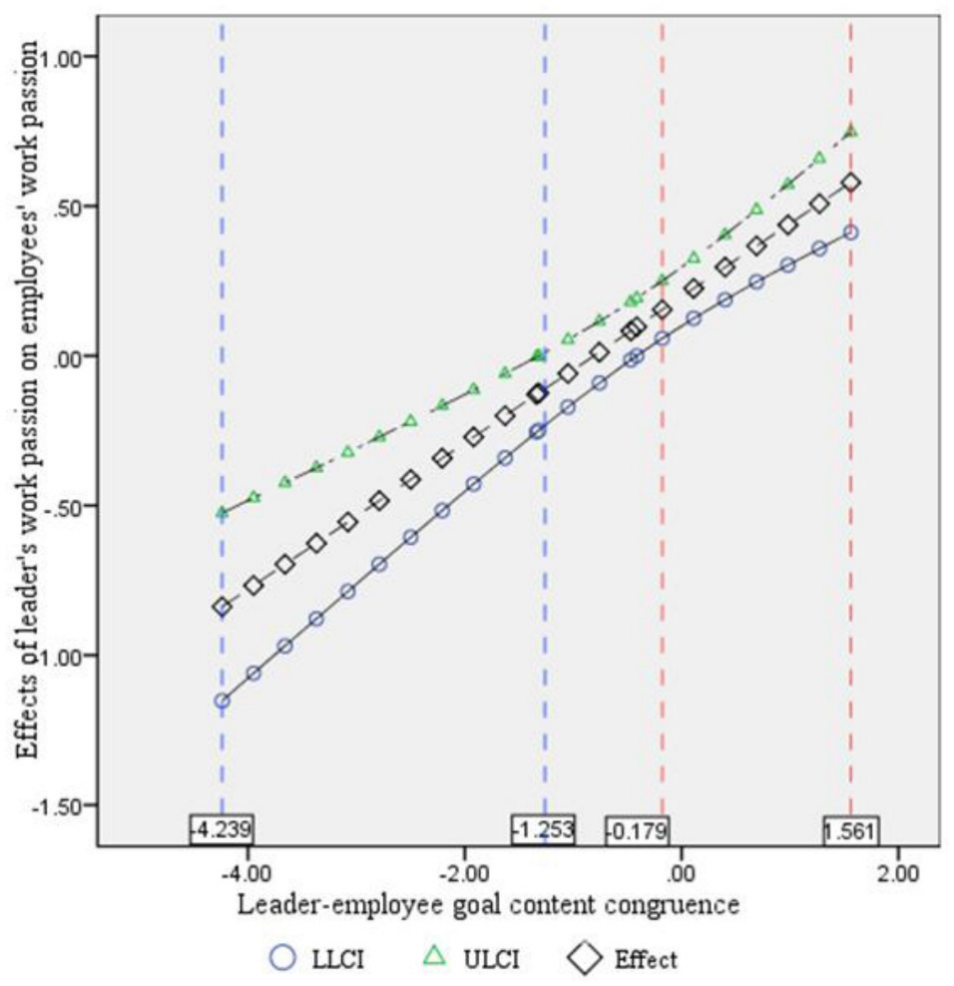
Effect of a leader's work passion on an employee's work passion with Johnson-Neyman confidence bands. The regions between blue lines and red lines are significant confidence regions. LLCI, lower level confidence interval and ULCI, upper level confidence interval.

Furthermore, we used the Johnson–Neyman technique to identify the regions in the range of the moderator variable where the effect of the leader's work passion on an employee's work passion is statistically significant and not significant (Preacher et al., 2006; Johnson, 2008; Hayes and Matthes, 2009; Prinzie et al., 2012). The results may help provide more preventive interventions and pointed suggestions. Figure 3 indicates that for the substitute value of leader–employee goal content congruence at the interval (−4.239, −1.253) and (−0.179, 1.561), a leader's work passion has a significant effect on an employee's work passion. That is, when the leader–employee goal congruence is at the low or middle level (interval from 0 to 0.179 and from 1.253 to 4.239), a leader's work passion may positively impact an employee's work passion, which is not consistent with the previous hypothesis (H3). As goal content contains intrinsic goals and extrinsic goals, they may have different effects on the relationship between a leader's work passion and employee's work passion. To explain this divergence, this research selected two types of extreme samples according to the different types of goal content (intrinsic goals and extrinsic goals): one sample is high intrinsic goal congruence with low extrinsic goal congruence, and the other sample is high extrinsic goal congruence with low intrinsic goal congruence. The results show that for the first group sample, a leader's work passion may positively significantly influence an employee's work passion, and the mediating effect of emotional contagion is significant (Table 5). However, for the second group sample, a leader's work passion does not influence an employee's work passion, and the mediating effect of emotional contagion is not significant (Table 6).

**Table 5:** Regression results for testing mediation role of emotional contagion (for high intrinsic goal congruence with low extrinsic goal congruence samples).

**Variables and statistic**	**EC**	**EWP**	
	**Model 2**	**Model 1**	**Model 3**
Constant	3.620^**^	1.735	−0.046
Gender (L)	0.019	−0.162	−0.171
Age (L)	−0.036	0.015	0.033
Tenure (L)	0.066	0.071	0.039
Education (L)	−0.162	−0.097	−0.017
Job position (L)	0.202	−0.311	−0.411
Gender (E)	−0.041	0.171	0.191
Age (E)	−0.001	−0.020	−0.019
Tenure (E)	0.016	−0.016	−0.024
Education (E)	0.104	−0.189	1.245
Job position (E)	−0.085	1.203	0.356
LWP	0.205^*^	0.457^**^	0.356^*^
EC			0.492^*^
*F*	0.938	1.404	1.932
*R*^2^	0.163	0.226	0.308

*N = 65, LWP, Leader's work passion; EC, Emotional contagion; EWP, Employee's work passion*.

** *p ≤ 0.01*,

* *p ≤ 0.05*.

**Table 6:** Regression results for testing mediation role of emotional contagion (for high extrinsic goal congruence with low intrinsic goal congruence samples).

**Variables and statistics**	**EWP**	**EC**
	**Model 1**	**Model 2**
Constant	3.099	3.567^*^
Gender (L)	0.222	−0.254
Age (L)	−0.008	−0.027
Tenure (L)	0.036	0.049
Education (L)	0.046	0.049
Job position (L)	−0.330	0.349
Gender (E)	−0.108	−0.356
Age (E)	−0.035	0.016
Tenure (E)	0.031	−0.020
Education (E)	0.225	0.002
Job position (E)	1.048	−0.331
LWP	0.233	0.098
*F*	1.129	0.554
*R*^2^	0.232	0.129

*N = 53, LWP, Leader's work passion; EC, Emotional contagion; EWP, Employee's work passion*.

* *p ≤ 0.05*.

## Discussion

This research has constructed a holistic model to explore whether and how a leader's work passion is transferred to employees. The results suggest that a leader's work passion may be transferred to employees via emotional contagion. Moreover, this process is positively moderated by leader–employee goal content congruence, particularly by leader–employee intrinsic goal content congruence. These findings have both theoretical and practical contributions.

### Theoretical contributions

First, this research has identified a positive association between a leader's work passion and employee's work passion, thereby extending work passion research to social network patterns. The majority of studies in work passion emphasize what and how individuals' work passion influences their own psychological state and behaviors, such as well-being (Zigarmi et al., 2009; Birkeland, 2014), job satisfaction, interpersonal relationships (Carbonneau et al., 2008; Curran et al., 2015), work performance (Ho et al., 2011; Donahue et al., 2012; Bélanger et al., 2013), organizational citizenship behavior (Perrewé et al., 2014; Astakhova, 2015), and organizational commitment (Permarupan et al., 2013). Limited studies have focused on the effects of an individual's work passion on others (Perrewé et al., 2014). This research has demonstrated the transference of work passion between leaders and employees. This finding inspires us to examine the phenomenon and mechanism of work passion transference among organizational members as well as consider whether work passion in an organization affects the work passion of individuals from other organizations. And it may lead to a combination between a social network technique and organizational psychology research.

Second, this research has confirmed the mediating effect of emotional contagion between a leader's work passion and employee's work passion, which provides empirical support for the conceptual model of entrepreneurial passion contagion. The entrepreneurial passion contagion model proposed by Cardon (2008) argues that passionate entrepreneurs are contagious. They display their positive emotions strongly and frequently, which motivates employees' positive affect and strengthens their identity with their work, which is work passion (Cardon, 2008). This model was applied only to entrepreneurial passion, and it remains theoretical without an empirical test. Our research applies the entrepreneurial passion contagion model to a general work context, thus enlarging its application and providing empirical support. Moreover, our results provide evidence that entrepreneurial passion and work passion may have the same structure (Li and Yu, 2013). Future research on entrepreneurial passion could refer to the research achievements in work passion to explore the source and mechanism of entrepreneurial passion.

Third, this research has constructed a preliminary holistic model of work passion contagion by the addition of leader–employee goal content congruence as a moderator, which is a beneficial supplement to the emotional contagion mechanism from both the leader and employee perspectives. Emotional contagion mainly depends on three factors: individual characteristics, situational factors, and interpersonal relations, such as gender (Doherty et al., 2006), individual traits (Zelenski and Larsen, 1999), power distance (Hsee et al., 1990), and relational factors (Van Orden and Joiner, 2006). As individual characteristics and situational factors (positions or rank level) are relatively stable, it is difficult to induce emotional contagion from these two aspects. Goal content is the root factor that affects interpersonal relations in work settings because it determines what individuals choose. When leader–employee goal content congruence is high, emotional contagion is more likely to occur. Because emotional contagion is a two-way interactive process, the consideration of factors from both leaders and employees is more practical.

In contrast, the subsequent research has resulted in more striking conclusions using the Johnson–Neyman technique to analyze the moderating effect of leader–employee goal content congruence. The results show that when leader–employee goal content congruence is at the middle level rather than the highest level, the effect of a leader's work passion on employee's work passion via emotional contagion is significant. After analyzing the intrinsic and extrinsic goals, respectively, we determined that when the leader–employee intrinsic goal congruence is high, and extrinsic goal congruence is low, the mediating effect is supported. However, when the leader–employee extrinsic goal congruence is high, and intrinsic goal congruence is low, all hypotheses are rejected. The reasons for this finding may be that intrinsic goal content is an orientation toward personal growth, including close relationships, community, self-acceptance, and physical fitness aspects (Broeck et al., 2008; Vansteenkiste et al., 2010). When both leaders and employees have intrinsic goal content orientations, they have more cooperative behaviors, and the transference of passion is smoother. In contrast, an extrinsic goal is an orientation toward greater wealth, fame, and image (Broeck et al., 2008; Vansteenkiste et al., 2010). Individuals with extrinsic goal content orientation have poor personal relationships, less cooperative behaviors, more prejudice, and more social aggression (Vansteenkiste et al., 2006). Therefore, when both leaders and employees have extrinsic goal content orientations, they have more potential competitive relations to achieve their own goals. Consequently, it is difficult to transfer passion. In summary, this finding provides a clearer boundary condition for the work passion contagion mechanism.

### Practical contributions

First, this research provides a novel approach to foster employees' work passion from the leader perspective. The results show that the immediate leader is a source of an employee's work passion. Influenced by Confucianism, Chinese leaders are not always good at displaying their emotions. Therefore, leaders should not only have work passion but should also display passion positively. Leaders should make their employees feel energized and find meaning in their work, make them internalize the meaning of their work into their self-identification, and help grow their work passion. Moreover, the organization must improve managers' leadership and stimulate employees' work passion.

Second, this study provides a solution from the emotional contagion view for leaders to evoke employees' work passion. Namely, affective motivation is more effective than monetary motivation in driving employees' work passion. According to social exchange theory, if an organization provides employees with more monetary motivation, employees will repay the organization with the same outcomes (Cropanzano and Mitchell, 2005), which means that improvements in salary and welfare may increase only employees' time and energy invest in work and their work performance; however, they cannot make employees identify with the meaning of their work. The identification of work and positive affect are the essential components of work passion. Thus, monetary rewards cannot inspire employees' work passion. In contrast, passionate leaders have positive expectations of organizations and strong intentions to communicate with employees, which are inspiring psychological and behavioral signals for employees. As a result, employees will make positive appraisals toward their work and organization, recognize the meaning of their work, have a desire to work, and eventually become passionate about their work (Zigarmi et al., 2011).

Third, leader–employee goal content congruence provides a preventive intervention for transferring work passion, particularly in Chinese organizations. For the Chinese social context, interpersonal relations (Guanxi) may be crucial factors that influence emotional contagion. As our discussion is related to the workplace, we should identify the factors that influence workplace relations from both of individuals in a relation. Moreover, goal content is the root factor of behavioral choice. When individuals have similar goal contents, they may have similar choices, and their interpersonal relations are closer; as a result, emotional contagion easily occurs. Therefore, organizations should consider the congruence of leaders' and employees' goal contents during personnel recruitment and allocation, thereby creating a harmonious and positive atmosphere.

## Limitations and suggestions for future research

This research has achieved valuable results; however, several limitations are worth noting. *First*, this research focused on the micro factors that influence work passion, whereas the organizational environment and job characteristics were not considered. According to the Affective Events Theory, the organizational environment may arouse employees' affective reactions through work events, thus positively influencing employees' work attitudes and behaviors (Weiss and Cropanzano, 1996). Zigarmi et al. (2011) also note that work passion originates from the cognitive and affective appraisals of various job and organizational situations. If these appraisals are positive and persistent, employees will have work passion. The organizational environment and job characteristics are the key factors that influence employees' appraisals. Therefore, future research should discuss the effects of different organizational environments and job characteristics on work passion.

*Second*, although this research constructed a moderated mediation model and analyzed the holistic process of work passion transference with the SPSS-PROCESS technique, it was a cross-sectional study, and there were limitations in explaining the causal relationship. Future research could design a longitudinal study and introduce the dynamic computational model to better identify the transference process from a leader's work passion to an employee's work passion.

*Third*, this research used Euclidean distance to represent the congruence of leaders' and employees' goal contents. Further research can refer to the social network theory to determine which nodes have closer relationships and which nodes are at the core locations in an organization. This would be a more visible and direct approach to reflect the process of work passion transference.

Fourth, this research pays attention to whether a leader's work passion can be transferred to employees, and how to transfer it. It hasn't discussed the different impacts of two types of leader's work passion (harmonious passion and obsessive passion) on employees. Future research can explore the possible different effects of two kinds of work passion and different moderating role of intrinsic goals and extrinsic goals.

Finally, all the samples are from China, and we haven't considered the cultural difference. Therefore, the external validity of our findings may not be accurate. Future studies should replicate our model using cross-national samples.

## Author contributions

All authors (JL, JZ, and ZY) substantially contributed to the research concept and design. JL predominantly contributed to drafting the manuscript and data analysis. JZ and ZY predominantly contributed to the acquisition of data. JL and JZ repeatedly revised and refined the content of the manuscript. All authors (JL, JZ, and ZY) contributed to the final approval of the version to be published. All authors (JL, JZ, and ZY) were accountable for all aspects of work in ensuring that questions related to the accuracy or integrity of any part of the work were appropriately investigated and resolved.

### Conflict of interest statement

The authors declare that the research was conducted in the absence of any commercial or financial relationships that could be construed as a potential conflict of interest.
